# Against All Odds: Education in Germany Coping with Covid-19

**DOI:** 10.1007/s42438-020-00130-7

**Published:** 2020-05-04

**Authors:** Michael Kerres

**Affiliations:** Universität Duisburg-Essen, Essen, Germany

**Keywords:** Educational Technology, Digital Change, Culture, Germany, Covid-19, Sociology

## High-Tech Innovators, Ed-Tech Laggards

Great times for learning technologists, one might think, in times of Covid-19! Out of a sudden, teachers are looking for digital tools to deliver learning materials to their students and organize communication within their classes. No managerial strategies, no teacher training, no debates on technological design or politics, no arguments about the pros and cons—we just do it. Worldwide, the use of technology in all fields of education is at a historical high. In Germany, though, we face a special situation. Germany is a world-leading developer and producer of high-tech products in many domains. And while the medical sector seems relatively well equipped to face the epidemic, educational system seems to be lagging in the use of digital technology for teaching and learning.

Ninety percent of German schools and universities are public institutions (Statistisches Bundesamt 2020), and Germany’s educational system heavily relies on autonomy of its 16 states (*Länder*). Robust data on the spread of digital infrastructure in education is not available (cf. Eickelmann et al. 2019), yet schools have been struggling with planning and implementing basic digital services for years. Only recently, schools have started to provide teachers with email accounts, web servers, and other learning technologies (News4Teachers 2019). In early 2019, the federal government decided to invest 5 billion euro for proliferating digital technologies in secondary schools (Bundesministerium für Bildung und Forschung 2020). But most school districts have not been able to spend the money so far, due to complex bureaucratic procedures that precede expenditure, and the need to first develop sound and aligned pedagogical concepts for the use of technology.

Given its economic strength, why is Germany so far behind? In Germany, digital technology in education is a highly debated topic. For decades, emotional discussions have been centering around the usefulness of computers in education. A German book written by a physician is receiving high attention in the public debate and explains—with references to brain science—that computers in schools lead to poorer performances, causing addiction and obesity (Spitzer 2012). Some parents have long been protesting, for example, against the installation of wireless LAN in schools, arguing for the negative effects of radio waves on the health of their children. The system of private Steiner Schools is successfully advertising their schools as non-digital for younger children (Freie Hochschule Stuttgart 2019).

## Constitutional Freedom of Teaching

German federal regulations restrict the adoption of software that is successfully used in education in other parts of the world. Teachers are strictly banned from using cloud services, social platforms, micro-blogs, or document sharing tools that are hosted outside of the EU, because of these technologies’ lack of (full) compliance with EU standards for privacy and data protection, telemetric practices, and the imponderables of data leaving EU territory. Germany proudly has world’s possibly strictest privacy and information protection legislation. The general ruling is not based on the aim of ‘securing’ data access and transport, but on the idea of *Datensparsamkeit* which could be translated as ‘data minimalism’ or ‘data austerity’ (Fowler 2013): the less data you store, the less data can be misused. While in some countries data is perceived as the ‘new oil,’ as a resource to run new businesses, *Datensparsamkeit* suggests that the best data is no data at all. Unsurprisingly, ambitious field research in learning analytics, which is often based on the exploitation of private data (Prinsloo and Slade 2014; Williamson 2017), is scarce. The exception that proves the rule is research on ‘trusted learning analytics’ carried out by Hendrick Drachsler’s group (2016).

Current school solutions are typically based on open source products, operated by states or regional school boards, on servers situated within national (or EU) boundaries. In order to avoid usage of software applications that could make data openly available to USA or other nations’ intelligence agencies, some states provide EU-compliant alternatives for document sharing and repository services. German universities have established a cooperative company, HIS Hochschul Informations System eG, Hannover, to develop administrative software solutions that operate according to EU data protection and privacy regulations. Ironically, in early 2020, a bug was encountered within the jointly developed student information system that was able to reveal personal data of all (!) students from nearly all public universities in Germany (Tremmel 2020). Digital textbooks need approval by state authorities and are hardly available. Due to inferior school infrastructure, book publishers are reluctant to invest in a somewhat unsure future—leaving not just production, but also development of expertise, to others.

Contradictions between Germany’s success in producing cutting-edge technology, and German caution when it comes to using digital technology in daily educational routines, are a fascinating research topic in their own right. Very briefly, they could be related to early nineteenth century Romanticism and its skeptical attitude towards technology at large. Furthermore, they could be associated with German more recent experiences of surveillance. Most Germans know (of) a person that has suffered either from the fascist regime 1933–1945 or from the communist regime 1945–1989 (in eastern part of Germany). Both regimes heavily relied on mass surveillance and total control of public opinion, which was especially prominent in broadcasting, newspapers, schools, and universities. For Germans, misuse of information is not an imagined danger at the horizon but a vivid experience reported by older generations. Mass murder of Jewish people was organized using a tabulator machine from German branch of IBM called Dehomag (Black 2012); population census used punch cards, where the infamous ‘column 22’ indicated a person with Jewish background (Luebke and Milton 1994). Germans’ ambivalent relationship to technology cuts across many different aspects of German culture, including philological and educational heritage. Its citizens may be successful engineers, yet it is hoped that they will also become well-rounded persons through *Bildung*.

For educators and educational (technology) researchers, the Covid-19 crisis rapidly opens new questions and develops new perspectives. Suddenly, and against earlier resistance against digital teaching and learning, teachers experience a steep learning curve while they implement all sorts of digital tools and materials in their work. They are caught by surprise when they find out that their university does not have a conference tool to communicate with more than 25 students at the same time, or that their university has limited student access licenses to online library materials. Their hectic attempts at compensation are aggravated by the ‘no-use’ policy for software hosted outside the EU or non-compliant with the European data protection regulations (GDPR). A solid video conferencing software serving, for example, 30,000 students at a larger university, does not seem easily available from an EU-compliant supplier. To stress that these rushed activities should not be equated with e-learning, distance education, or another form of carefully planned and administered online learning experience, Germany has quickly picked up the term ‘remote teaching’ (Hodges et al. 2020).

In response to its recent history, Germany is one the few world’s nations where freedom of research and teaching is codified in the constitution (Bundesministerium der Justiz und für Verbraucherschutz 2020: Art 5). Amongst other things, this constitutional right implies that teachers can freely choose whether they want to use digital technology. During the Covid-19 crisis, discussions have emerged about the consequence of ‘remote teaching’ pointing to access inequities. While many teachers have heavily invested in remote teaching with digital tools, some teachers have demanded the immediate stop of the promotion of digital tools. For instance, a group of scholars from the well-respected Ludwig Maximilian University in Munich, with the support of many other professors from other universities, demanded the cancelation of studies for the summer semester (#nichtsemester 2020).

## Educational Leadership and Digital Change Under Pressure

Besides anecdotical reports, we do not have reliable data about numbers of (university) teachers who have been using technology for ‘remote teaching’ during the crises and about their success. It seems that we probably will not have this data in the near future, because planned or approved projects on the topic do not seem to be in sight. Many teachers have now quickly, even if non-systematically, developed their digital competencies. After the Covid-19 crisis is gone, will they immediately return to earlier strong preference for face-to-face teaching or will they start moving towards online education? (see Weller 2020) Answers to this question will depend on teacher and student experiences during the crises. Will they perceive the remote learning experience as an ambivalent but feasible alternative? Will they remember remote teaching as a traumatic episode that needs to be quickly left behind? These questions will probably inspire a lot of postdigital research after the crisis. As Jandrić (2020) has pointed out, this historical moment challenges many common positions in research and education and calls for a lot of rethinking in and for the future.

At the learning lab of University Duisburg-Essen, we are studying practices of change in educational institutions (Kerres and Waffner 2019). Confronted with the crisis where teachers have turned to digital technology with little or no organizational support, we are now seriously rethinking our models of ‘digital change.’ We are questioning previous measures: digital strategies, policies, projects, incentives, and support. Will current changes, experienced by individual teachers, affect their organizations at large? Before the Covid-19 crisis, research on educational leadership and digital change has not paid much attention to reactions of educational organizations under extreme external pressure (e.g., Davis 2017). All organizations react to external conditions such as market shifts, but how will they integrate personal and collective experiences of this state of emergency? Answers to these questions will depend, amongst other factors, on social constructions and meaning-making of various stakeholders. And then, for postdigital research, it will be important to understand these developments against the backdrop of cultural conditions and its historical foundations.
